# Automated Rib Fracture Detection on Chest X-Ray Using Contrastive Learning

**DOI:** 10.1007/s10278-023-00868-z

**Published:** 2023-07-05

**Authors:** Hongbiao Sun, Xiang Wang, Zheren Li, Aie Liu, Shaochun Xu, Qinling Jiang, Qingchu Li, Zhong Xue, Jing Gong, Lei Chen, Yi Xiao, Shiyuan Liu

**Affiliations:** 1 Department of Radiology, Shanghai Changzheng Hospital, Navy Medical University, No.415, Fengyang Road, Huangpu District, Shanghai, 200003 China; 2 Institute for Medical Imaging Technology, School of Biomedical Engineering, Shanghai Jiao Tong University, Shanghai, 200030 China; 3 Shanghai United Imaging Intelligence Co., Ltd., No.701, Yunjin Road, Xuhui District, Shanghai, 200232 China; 4 Departments of Radiology, Changhai Hospital, Navy Medical University, Shanghai, 200433 China

**Keywords:** Rib fracture, Deep learning, X-ray, CT images, Multicenter

## Abstract

To develop a deep learning-based model for detecting rib fractures on chest X-Ray and to evaluate its performance based on a multicenter study. Chest digital radiography (DR) images from 18,631 subjects were used for the training, testing, and validation of the deep learning fracture detection model. We first built a pretrained model, a simple framework for contrastive learning of visual representations (simCLR), using contrastive learning with the training set. Then, simCLR was used as the backbone for a fully convolutional one-stage (FCOS) objective detection network to identify rib fractures from chest X-ray images. The detection performance of the network for four different types of rib fractures was evaluated using the testing set. A total of 127 images from Data-CZ and 109 images from Data-CH with the annotations for four types of rib fractures were used for evaluation. The results showed that for Data-CZ, the sensitivities of the detection model with no pretraining, pretrained ImageNet, and pretrained DR were 0.465, 0.735, and 0.822, respectively, and the average number of false positives per scan was five in all cases. For the Data-CH test set, the sensitivities of three different pretraining methods were 0.403, 0.655, and 0.748. In the identification of four fracture types, the detection model achieved the highest performance for displaced fractures, with sensitivities of 0.873 and 0.774 for the Data-CZ and Data-CH test sets, respectively, with 5 false positives per scan, followed by nondisplaced fractures, buckle fractures, and old fractures. A pretrained model can significantly improve the performance of the deep learning-based rib fracture detection based on X-ray images, which can reduce missed diagnoses and improve the diagnostic efficacy.

## Introduction

Rib fractures are common blunt chest traumas that occur in 20% of all cases of chest trauma and affect approximately 40% of patients who suffer from severe chest trauma [1, 2]. The clinical symptoms of patients with rib fractures include localized pain, abnormal breathing, and skin bruising. The number of rib fracture locations and the type of fracture can indicate the severity of trauma and can be used to predict complications and mortality rates [3–5]. Conventional imaging examinations for identifying and classifying rib fractures are based on chest X-ray and CT. According to the imaging modality selection criteria established by the American College of Radiologists, an X-ray (or DR) is usually obtained to initially diagnose trauma patients because it is convenient and inexpensive [6]. However, missed diagnosis and misdiagnosis of rib fractures often occur due to ambiguity caused by multiple overlapping anatomical structures coupled with different locations and sizes of fractures in X-ray; moreover, image reading requires experience and training for radiologists [7, 8]. Therefore, to reduce miss- and misdiagnosis, an automatic rib fracture detection and localization method is highly desirable to assist radiologists in reading chest X-ray images.

In the literature, deep learning-based (DL) models have demonstrated effectiveness in helping radiologists quickly and precisely identify rib fractures in CT images [9–11]. For X-ray images, several studies have illustrated unprecedented success in applying DL-based models to detect suspicious fractures, such as hip fractures [12], mandibular fractures [13], scaphoid fractures [14], and femoral neck fractures [15]. In one study [16], a DL-based model of chest radiography was established to identify four types of features (fracture, pneumothorax, nodule, and opacity), and the sensitivity and specificity for fracture detection based on the public dataset ChestX-Ray14 were 43.1% and 92.8%, respectively. However, many delineated images are needed to train DL-based models. To address this problem, an automatic rib fracture recognition algorithm was designed using mixed supervised learning while considering the spatial information from class activation maps [17]. The experimental results showed that the recognition algorithm outperformed other methods by annotating 20% of the positive samples in 10,966 images.

Moreover, to overcome the weak prominence of fractures in chest X-ray images, a hybrid supervised learning model was established to provide fracture classification decisions and change image interpretation [17]. This model incorporated partial correlation decoupling and example separation enhancement strategies to accurately detect fracture regions [17]. Nevertheless, compared to CT, it is still challenging for deep learning models to achieve radiologist-level merit for rib fracture detection on chest X-ray.

In this study, we compare the performance of different fully convolutional one-stage (FCOS) objective detection pretraining methods [18], such as a model with no pretraining (no pretraining), a model pretrained with ImageNet data (pretrained ImageNet), and a model pretrained with DR images (pretrained DR), to validate a deep learning-based model for detecting rib fractures in X-ray images.

## Materials and Methods

This study was performed with adherence to the principles of the Declaration of Helsinki. Approval was granted by the Ethics Committee of Shanghai Changzheng Hospital (No.2022SL071), and the requirement for informed consent of patients was waived due to the retrospective nature of the analysis and the anonymity of the data.

### Datasets

A total of 18,631 chest DR scans (anteroposterior or posteroanterior) were collected for the pretraining, training, and testing of the rib fracture detection model, with all images unique. The datasets used for pretraining included a public dataset, ChestX-Ray14 [19], and a dataset collected from multiple hospitals, denoted as DR-data. ChestX-Ray14 consists of 16,253 radiographs in a posteroanterior projection with fracture cases (7424 images) and nonfracture cases (8829), and 1842 cases in DR-data are fracture cases based on the corresponding radiological reports. All these data were labelled as 1 for fracture (with bounding boxes for each fracture) and 0 for nonfracture.

For training and testing, we collected two datasets, Data-CZ and Data-CH. Data-CZ consists of 427 images/cases (1388 fractures) from Shanghai Changzheng Hospital and was used for training (300 fracture cases) and testing (72 fracture and 55 no-fracture cases). Data-CH includes 109 paired chest DR and CT images (290 fractures) from Changhai Hospital and was used for testing. All the fracture cases were also annotated into four types, namely F0, displaced fractures; F1, nondisplaced fractures; F2, buckle fractures; and F3, old fractures [10]. Each fracture in the ground-truth data was considered in the evaluation of the detection models.

Details of the pretraining, training, and testing datasets are listed in Table 1. This retrospective study was approved by the institutional review boards of the respective hospitals, all patient information was anonymized, and informed consent forms were waived. The inclusion criteria for our privately collected data were as follows: (a) age of 18 years or older, (b) DR or CT for acute chest trauma within 2 weeks, and (c) complete DR and CT image data. The exclusion criteria were as follows: (a) images with artifacts caused by malposition, respiratory motion, etc.; (b) patients with post-thoracic spine and rib surgery; (c) pathological fractures or other nonfracture lesions of the ribs; and (d) thoracic deformities. All the data were examined by two radiologists from Shanghai Changzheng Hospital (5 years of experience and 7 years of experience), by referring to the corresponding CT images. In cases when a consensus was not reached, a third expert (12 years of experience) further evaluated the images. Finally, the fracture sites and fracture types were marked on the images by using an internal labelling tool.

**Table 1:** The datasets used for pretraining, training, and testing of the fracture detection model. The pretraining data are labelled to indicate a fracture or no fracture, and the training and testing data are labelled with different fracture types

**Pretraining data**	Fracture status	No. of patients	Facture label				
ChestX-ray14	Fracture	7424	Fracture (1) bounding box				
	No fracture	8829	Normal (0)				
DR-data	Fracture	1842	Fracture (1) bounding box				
**Training/testing data**	Types	Patients	F0	F1	F2	F3	Total
Training Data-CZ	Fracture	300	683	239	181	8	1111
Testing Data-CZ	Fracture	72	158	74	40	3	275
	No fracture	55	/	/	/	/	/
Testing Data-CH	Fracture	109	168	54	60	8	290
	Total	18,631					

*F0*, displaced fractures; *F1*, nondisplaced fractures; *F2*, buckle fractures; *F3*, old fractures

**Fig. 1:**
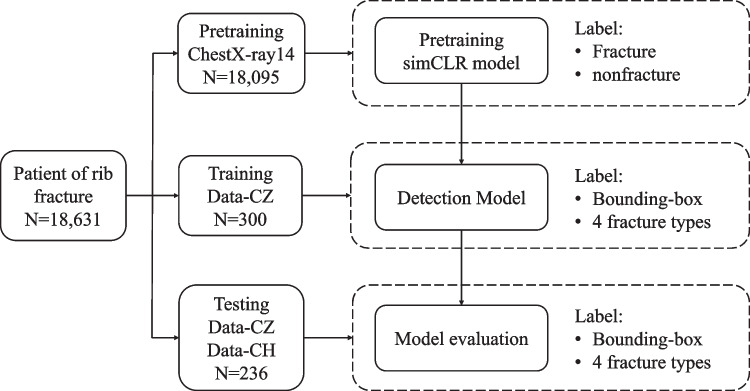
Schematics of labelled data used for the pretraining, training, and testing of the deep learning models

Accurate fracture annotations are critical for training and testing DL-based models. In fact, for DR images, neither the hierarchy annotation strategy nor the majority-voting approach can prevent interreader variability and mislabelling. Due to the ambiguity of DR images, and compared to DR images, CT images provide clearer visualization of bone fractures. Therefore, we double-checked the ground truth data, the bounding box drawn on X-ray images, by referring to the corresponding CT images. The corresponding locations of the fractures between 2D chest X-ray (2D bounding box) and 3D CT images (3D volume) were checked and confirmed by a radiologist. The details of the annotations for each stage of model development are given in Fig. 1.

### Pretrained Detection Model simCLR

Because the training dataset with four types of rib fractures labelled is limited, we first built a pretraining model for fracture detection using public X-ray datasets, that included fracture labels (0 indicating no fracture, 1 indicating a fracture) but lacked location and type annotations. The model derived from contrastive learning was based on a simple framework, simCLR, which was proposed by Hinton’s group [20] to improve the performance of DL-based models with limited labelled data. simCLR serves as a pretrained model for various downstream tasks such as classification and detection [20]. In this work, we first use simCLR to extract deep features from public datasets (ImageNet and ChestX-Ray14) and then transfer the pretrained network to the downstream tasks of rib fracture detection from chest X-ray images.

**Fig. 2:**
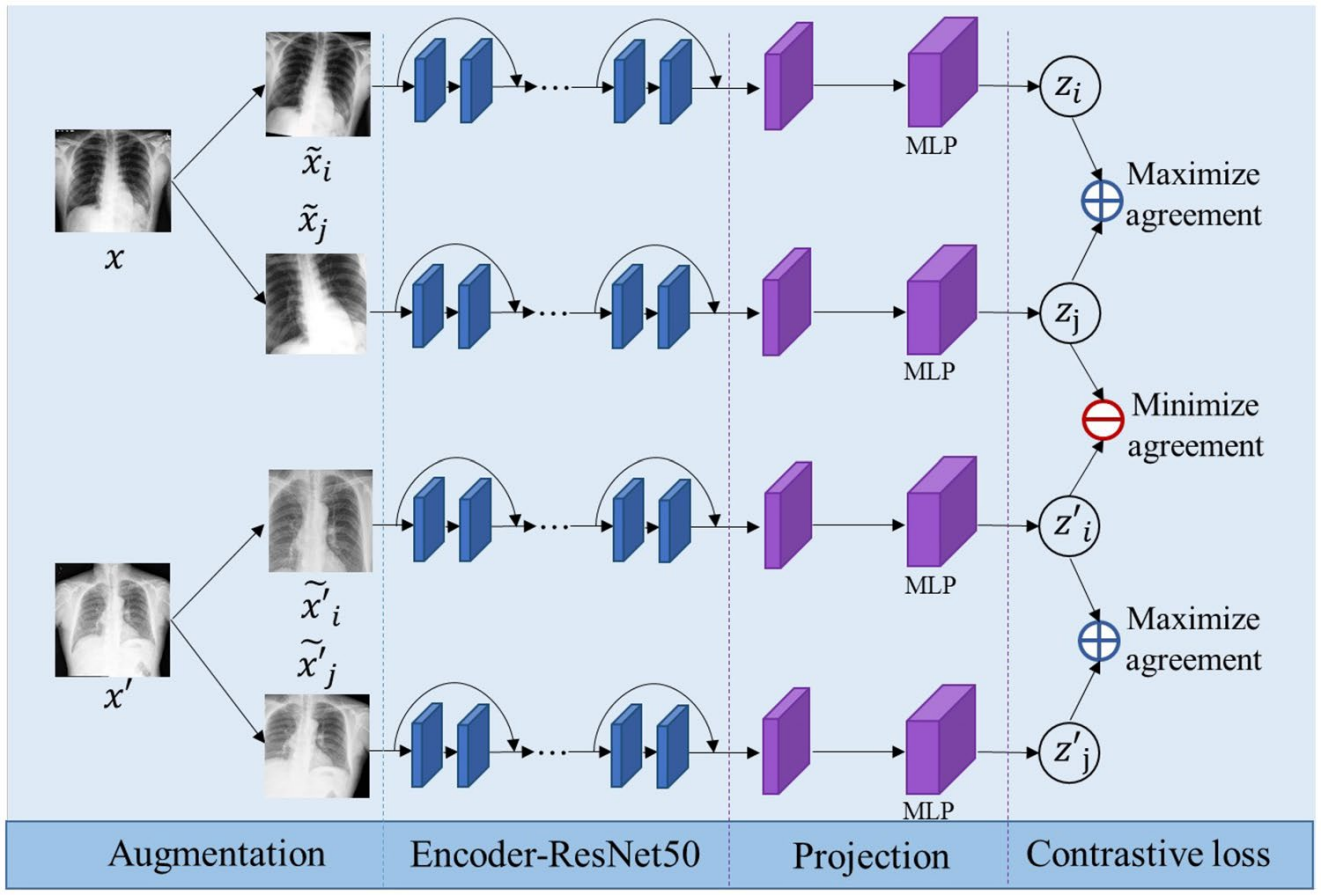
Detailed illustration of self-supervised pretraining for rib fracture detection from the chest X-ray images

One of the mainstream approaches to learn visual representations without supervision is a discriminative algorithm based on contrastive learning. Contrastive learning is a self-supervised learning method, that trains an encoder to yield an effective image representation without labelling. simCLR [20] learns reliable representations in the feature space by maximizing agreement between different augmentations. Specifically, each sample is randomly processed by two separate data augmentation operators, such as cropping, rotation, and style transfer. As a result, given a mini-batch of $N$ images, $2N$ samples can be generated. In the augmented mini-batch, two augmented images from the same sample are grouped as a positive pair $(i,j)$, whereas the other $2(N-1)$ augmented images in the mini-batch are grouped as negative examples. Contrastive learning is driven by contrastive loss, and the goal is to maximize the agreement among positive pairs while minimizing the agreement between positive and negative pairs. Therefore, the learned features are generalized and robust to diversifying operations. A detailed illustration of self-supervised pretraining for rib fracture detection from chest X-ray images is shown in Fig. 2.

The contrastive loss for a positive pair is defined as follows:

1 $$\mathrm{loss}(i,j)=-\mathrm{log}\frac{\mathrm{exp}(\mathrm{sim}({z}_{i},{z}_{j})/\tau )}{{\sum }_{k=1}^{2N}{1}_{[k\ne i]}\mathrm{exp}(\mathrm{sim}({z}_{i},{z}_{j})/\tau )},$$

where $\mathrm{sim}({z}_{i},{z}_{j})$ is the dot product (cosine similarity) between the extracted features ${z}_{i}$ and ${z}_{j}$. ${1}_{[k\ne i]}\in \left\{0, 1\right\}$ is the indicator function, which equals 1 when $\mathrm{k}\ne \mathrm{i}$. $\tau$ is a temperature parameter, and the network loss is defined as follows:

2 $$\mathrm{LOSS}=\frac{1}{2N}{\sum }_{k=1}^{N}[\mathrm{loss}\left(2k-1, 2k\right)+\mathrm{loss}(2k, 2k-1)].$$

An original image $x$ is transformed into images ${\widetilde{x}}_{i}$ and ${\widetilde{x}}_{j}$ via two random augmentations. Then, ${\widetilde{x}}_{i}$ and ${\widetilde{x}}_{j}$ are passed through an encoder network (ResNet50). The representations generated by the encoder network are then passed into a contrastive loss function that promotes similarity between representations ${z}_{i}$ and ${z}_{j}$. The trained model retains the base encoder network, and the 2-layer MLP projection head is removed as a pretrained model for FCOS objective detection.

### Fully Convolutional One-Stage Object Detection Model

**Fig. 3:**
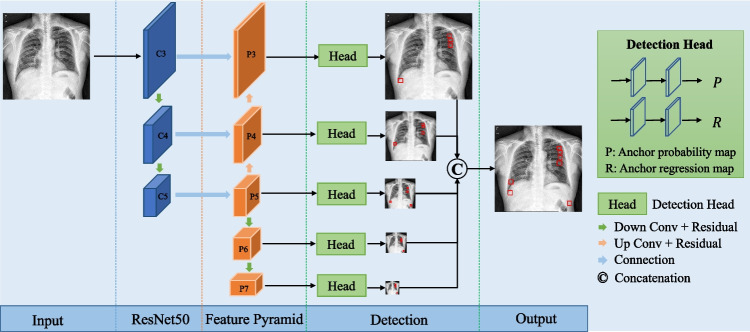
Network architecture of the FCOS objective detection model. The parameters of ResNet50 are initialized by pretrained models

In this study, a classic detection network, the FCOS objective detector [18], was applied to identify fractures in frontal chest X-rays. The FCOS objective detection approach involves a classic single-view detection network that is able to balance speed and performance and can handle a wide range of object detection tasks. With post-processing non-maximum suppression, the FCOS objective detection is anchor box free and completely eliminates complicated computations related to anchor boxes. The model also performs object detection in a per-pixel prediction fashion and avoids using all the hyperparameters related to anchor boxes. The pretrained model is used to initialize the parameters of the detection model. The derived pretrained model from contrastive learning is adopted as the backbone of the FCOS architecture for the realization of the downstream lesion detection tasks. Therefore, we demonstrate a highly simple and flexible detection framework for rib fracture identification, as shown in Fig. 3.

### Statistical Analysis

To evaluate the performance of the fracture detection algorithms, we calculated the sensitivity of fracture detection and summarized the free-response ROC (FROC) curves for each experiment. Referring to the rules of the LUNA challenge [21], the criterion to determine if a prediction result matches the annotation is based on whether the predicted box and the annotated box overlap each other. In this case, the detection result is considered a true positive (TP) if the intersection over union (IoU) is more than 30% [22]; otherwise, it is a false positive (FP) compared with any ground truth bounding boxes. On the other hand, if no predicted nodule matches an annotated nodule, the missed nodule is counted as a false negative (FN). Based on TP, FP, and FN, the sensitivity of the detection is given as $\mathrm{sensitivity}=\frac{\mathrm{TP}}{\mathrm{TP}+\mathrm{FN}}$. For the FROC curve, the *x*-axis represents the average number of FPs per scan, and the *y*-axis represents sensitivity. SPSS 25.0 software (version 25.0; SPSS Inc., Chicago, IL, USA) was used for statistical analysis. A paired-sample *t*-test was used to compare the sensitivity of detection for the three DL models. A *P* value <0.05 indicated a statistically significant difference.

**Fig. 4:**
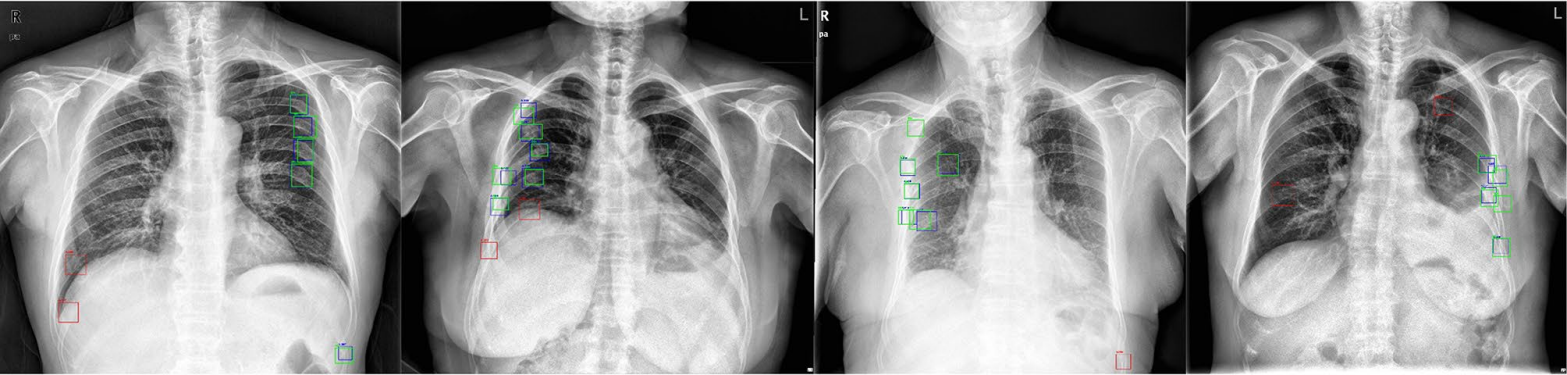
Illustrations of the rib fractures detected by pretrained DR + FCOS and manual annotations from Data-CZ. Green, ground truth; blue, true positive; Red, false positive

With the public datasets ImageNet and ChestX-ray14, we trained the pretrained ImageNet and pretrained DR models, respectively. The pretrained models were then used to initialize the parameters of the FCOS objective detection model. Herein, pretrained ImageNet means the pretraining model was built using the ImageNet dataset (1.2 million labelled images depicting 10,000+ object categories), and the pretrained-DR model was trained using chest DR scans from the dataset of ChestX-ray14 and data from hospitals. Then, the FCOS objective detection model was trained with 300 chest X-ray images, which includes 1111 fractures. Examples of rib fractures detected from Data-CZ with the proposed method are shown in Fig. 4.

## Results

### Performance of Rib Fracture Detection

**Fig. 5:**
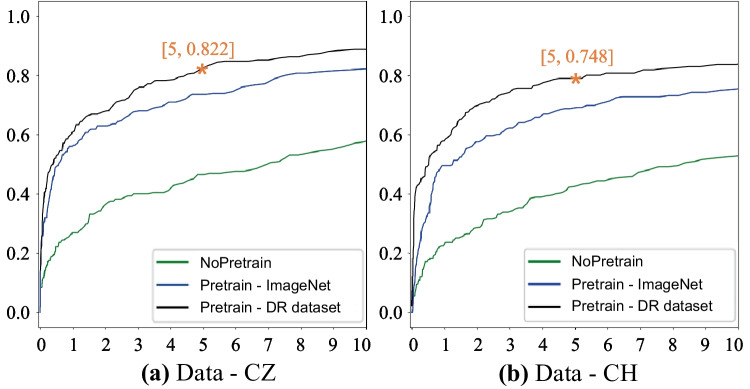
Quantitative comparison of FROC curves for no pretraining, pretrained ImageNet, and pretrained DR models in the ablation study based on Data-CZ collected from Shanghai Changzheng Hospital (**a**) and Data-CH collected from Changhai Hospital (**b**)

To evaluate the performance of the detection models with two pretrained models, we calculated the sensitivity in cases with different numbers of FPs, as shown in Table 2, and the FROC curve results are plotted in Fig. 5. Based on a compromise between sensitivity and the number of FPs, our model reached the best performance with five FPs per scan, which might be tolerable clinically. With the 127 chest DR scans in the testing dataset of Data-CZ, we performed an ablation study, and the detection sensitivities of the no pretraining, pretrained ImageNet, and pretrained DR models were 0.465, 0.735, and 0.822, respectively, as listed in Table 2, based on five FPs per scan. For 109 DR scans in Data-CH, the sensitivities of the three pretrained methods were 0.403, 0.655, and 0.748.

**Table 2:** Performance of the three models in sensitivity and ablation analyses with test images from Data-CZ and Data-CH

Testing set	Pretraining strategy	Sen@1	Sen@2	Sen@3	Sen@4	Sen@5
Data-CZ	No pretraining	0.269	0.360	0.400	0.411	0.465
	Pretrained ImageNet	0.564	0.629	0.680	0.709	0.735
	Pretrained DR	0.607	0.680	0.760	0.785	**0.822**
Data-CH	No pretraining	0.217	0.272	0.321	0.369	0.403
	Pretrained ImageNet	0.469	0.545	0.590	0.634	0.655
	Pretrained DR	0.548	0.662	0.703	0.734	**0.748**

*Sen@1*, detection sensitivity when the average FP per scan is 1; *Pretrained ImageNet*, pretrained using the ImageNet dataset; *Pretrained DR*, pretrained using chest DR scans

The FCOS objective detection model combined with the pretrained DR model achieved the best performance, and the sensitivity was 0.822 and 0.748 with five FPs for the test datasets Data-CZ and Data-CH, respectively; a comparison of these results with those of the no pretraining and pretrained ImageNet models is shown in Fig. 5. The detection performance of no pretraining and pretrained DR increased significantly from 0.465 to 0.822 (Data-CZ: *t*=−37.2, *P*=0.000) and from 0.403 to 0.748 (Data-CH: *t*=−32.8, *P*=0.000), respectively. The pretrained ImageNet model did not perform as well as pretrained DR (Data-CZ: *t*=−7.8, *P*=0.001, and Data-CH: *t*=−14.6, *P*=0.000).

### Detection Performance for Four Fracture Types

In the previous section, the detection model was initialized with the pretrained models and achieved significantly better performance than those without pretraining. Based on the detection results, we further analyzed the performance of the models in the detection of different fracture types. For the testing images of Data-CZ and Data-CH, the detection model achieved the highest performance for displaced fractures, with sensitivities of 0.873 and 0.774, respectively, with 5 FPs per scan, followed by nondisplaced fractures and buckle fractures. Old fractures were the most complicated type to detect, with sensitivities of 0.667 and 0.500, for Data-CZ and Data-CH, respectively. The details are shown in Table 3.

**Table 3:** Performance (sensitivity) of FCOS objective detection model for four fracture types. The parameters of the FCOS objective detection model were initialized using the pretrained DR model

Testing set	Fracture type	Sen@1	Sen@2	Sen@3	Sen@4	Sen@5
Data-CZ	F1	0.703	0.766	0.823	0.848	**0.873**
	F2	0.541	0.595	0.689	0.716	0.743
	F3	0.375	0.500	0.650	0.675	0.775
	F4	0.333	0.667	0.667	0.667	0.667
Data-CH	F1	0.607	0.720	0.738	0.768	**0.774**
	F2	0.519	0.667	0.722	0.722	0.759
	F3	0.450	0.517	0.617	0.683	0.700
	F4	0.250	0.500	0.500	0.500	0.500

*F1*, displaced fractures; *F2*, nondisplaced fractures; *F3*, buckle fractures; *F4*, old fractures; *Sen@1*, detection sensitivity when the average FP per scan is 1

**Fig. 6:**
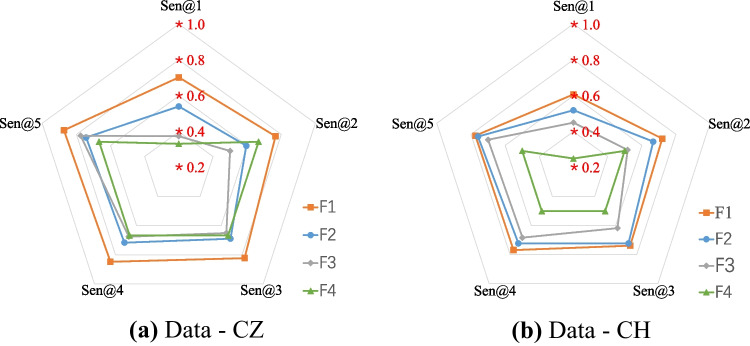
Radar map of sensitivities with different FP values (1, 2, 3, 4, and 5 FPs per scan) based on Data-CZ and Data-CH for various fracture types

In addition, a radar map (Fig. 6) clearly shows that the performance of the detection model was better for Data-CZ than for Data-CH. The fracture detection results indicated that the detection of displaced fractures was better than that of other fracture types at different FP values [1–4, and 5], followed by nondisplaced fractures and buckle fractures. Old fractures are the most challenging type to detect. The F1 performance was higher than others and had significant differences (Data-CZ F1 vs. F2 *P*=0.000, F1 vs. F3 *P*=0.007, F1 vs. F4 *P*=0.011, and Data-CH F1 vs. F2 *P*=0.032, F1 vs. F3 *P*=0.006, F1 vs. F4 *P*=0.000). For the other three fracture types, there was no significant difference for Data-CZ (all *P*>0.05), and there was a significant difference for Data-CH (F2 vs. F3 *P*=0.013, F2 vs. F4 *P*=0.000, F3 vs. F4 *P*=0.015).

### Radiologist Performance Using the DL Model

The performance of radiologists was evaluated with and without the DL model based on Data-CZ. The sensitivity for rib fractures diagnosed by the radiologist (SEN=0.731) was lower than that for the DL model (SEN=0.822), and the difference was significant (${\chi }^{2}=6.5,P=0.01$). The sensitivity of the radiologist was improved with the DL model (SEN=0.865) across all four fracture types compared to that in the case without the DL model, and the difference was significant (${\chi }^{2}=15.5,P=0.00$). With the help of the DL model, missing positive cases, such as nondisplaced fractures, were identified by the radiologist.

## Discussion

We developed deep learning models for rib fracture detection on chest X-ray and evaluated the diagnostic efficacy of the models with two testing datasets. Among the two pretrained models, for both datasets, the pretrained DR model performed best, yielding a sensitivity of 0.822 for the CZ dataset and 0.748 for the CH dataset at an average of five FPs, which is beyond our expectation and better than the results of previous related studies.

Rib fractures are common and severe injuries in chest trauma cases, and some patients have an obvious manifestation of compound injuries. The threat to patients increases in complicated and aggravated cases [8, 23]. Therefore, there is a need to diagnose and treat these conditions as early as possible. In addition, rib fracture has a high missed diagnosis rate. Cho et al. reported a missed diagnosis rate of up to 20.7% based on CT images [24]. The missed diagnosis rate for DR is even higher (up to 33–50%) [7, 25]. The missed diagnosis of fractures not only affects patient prognosis but also leads to adverse medicolegal disputes. Multilayer spiral CT can improve the accuracy of rib fracture diagnosis by reconstructing multilayer scan data in 3D. However, in the first instance of injury, an X-ray is still the primary diagnostic modality for rib fractures due to the low radiation dose and high convenience and affordability.

Anna Majkowska et al. applied a deep learning model to detect fracture lesions at multiple sites in DR, including clavicle, shoulder, rib, and spine fractures and spanned acute, subacute, and chronic conditions [16]. Compared to manual reading, this model demonstrated higher sensitivity for acute fracture identification (with an AUC of 0.86). Among the different fractures in DR, rib fractures are the most challenging to detect, as they are easily affected by postural and anatomical factors. Our model reduced false positives with high sensitivity and detected four different types of fractures simultaneously. We obtained a sensitivity of 43.1% and a specificity of 92.8% for the public dataset ChestX-Ray14. We believe that this was due to some limitations of X-ray results, such as heavy organ overlap artifacts, and the limited information in X-ray images, resulting in poor performance for the deep learning model.

Therefore, we used a more straightforward and flexible deep learning model, the classical FCOS objective detection network [18], to identify fractures in orthopantomograms of chest DR. The efficiency of this model was significantly improved for the CZ and CH datasets. The sensitivity for the CZ data was 0.607 and 0.822 for one and five FPs on average, respectively; these values were within the acceptable range. In contrast, the sensitivity for CH data was 0.548 and 0.748 for one and five FPs on average, respectively. The experimental results showed that a pretrained model can considerably enhance rib fracture detection. Furthermore, our deep learning model is highly generalizable and can be applied in different hospitals.

Furthermore, we analyzed the detection performance of the models for four different fracture types. The sensitivities were highest for displaced fractures at 0.873 and 0.774 for five FPs per scan based on the CZ and CH test sets, respectively, and these results were consistent with those in other research. Because nondisplaced fractures and cortical bone distortions do not involve significant displacement, it is challenging for clinicians to accurately identify these two types of fractures. However, our model achieved unprecedented success in detecting both types of fractures. In contrast, our model was less sensitive for old fracture detection. We believe the reason may be linked to the small size of the old fracture sample. Moreover, old fractures are characterized by mature calluses, no clear fracture lines, and a morphology similar to that of the surrounding healthy ribs. Therefore, it is difficult to distinguish them from surrounding healthy ribs. In the future, the model will be further optimized, and the accuracy will be further improved as the sample size increases. In addition, only rib fracture detection was considered in this study, and it is equally important for clavicle, scapula, and spine fracture detection [26, 27]. In addition, the detection of severe complications due to trauma, such as pneumothorax issues, atelectasis, and cardiac great vessel injury, should be considered in future models [28–31]. Risk prediction models could also be established by combining the clinical semantic features of patients (e.g., blood pressure and respiration) [32]. It is believed that future multitasking models will be more suitable in clinical application scenarios to further benefit patients.

There were some limitations in this study. First, this study was retrospective, and although various types of fractures were detected, clinical work with cortical bone distortion and old lesions may not be of significant clinical significance for emergency management. Thus, further important features will be detected according to clinical needs. Second, we did not compare the performance of our model with physician-based models in terms of key factors, such as clinical outcomes and the time required to obtain a diagnosis. Finally, the size of the validation set selected in our model was relatively small. Additional data should be obtained, and prospective studies should be subsequently performed to validate the convolutional neural network (CNN) model.

## Conclusion

A DL-based rib fracture detection model was proposed, which achieved good performance in detecting different rib fractures from chest X-ray based on multi-center/multi-parameter validation. The results illustrated that compared to traditional models, the DL model not only improved the diagnostic efficiency but also reduced the diagnostic time and eased the workload of radiologists. Therefore, it can be used to diagnose rib fractures from chest X-ray images with the assistance of artificial intelligence. In the future, we will study ways to incorporate clinical measures and medical images into 3D CNN architectures to achieve risk prediction for rib fracture patients.

## Data Availability

The data that support the findings of this study are available on request from the corresponding author. The data are not publicly available due to privacy or ethical restrictions.

## Abbreviations

DR: Digital radiography
simCLR: Simple framework for contrastive learning of visual representations
FCOS: Fully convolutional one-stage
DL: Deep learning
FROC: Free-response ROC
IoU: Intersection over union
CNN: Convolutional neural network
TP: True positive
FP: False positive
FN: False negative
